# *trans*-Bis(3-hy­droxy­pyridine-κ*N*)diiodidoplatinum(II) dimethyl sulfoxide disolvate

**DOI:** 10.1107/S1600536811015893

**Published:** 2011-05-07

**Authors:** Fazlul Huq, Muhammed Danish, Wojciech Starosta, Janusz Leciejewicz

**Affiliations:** a Sydney Medical School, The University of Sydney, Cumberland Campus, 75 East Street, Lidcombe, NSW 1825, Australia; b Department of Chemistry, University of Gujrat, Hafiz Hayat Campus, Gujrat 50700, Pakistan; c Institute of Nuclear Chemistry and Technology, ul.Dorodna 16, 03-195 Warszawa, Poland

## Abstract

In the title compound, [PtI_2_(C_5_H_5_NO)_2_]·2(CH_3_)_2_SO, the Pt^II^ ion lies on an inversion center and is coordinated in a slightly distorted square-planar environment by two *trans* iodide ligands and two pyridine N atoms. In the crystal, complex mol­ecules and solvent dimethyl sulfoxide mol­ecules are linked by inter­molecular O—H⋯O hydrogen bonds.

## Related literature

For the results of activity, cell uptake and DNA binding studies of some *trans*-planar platinum complexes, see: Farrell *et al.* (1992 ▶); Bierbach *et al.* (1999 ▶); Huq *et al.* (2004 ▶); Daghriri *et al.* (2004 ▶); Chowdhury *et al.* (2005 ▶). For the structure of *trans*-dichloridoplatinum(II), see: Beusichem & Farrell (1992 ▶).
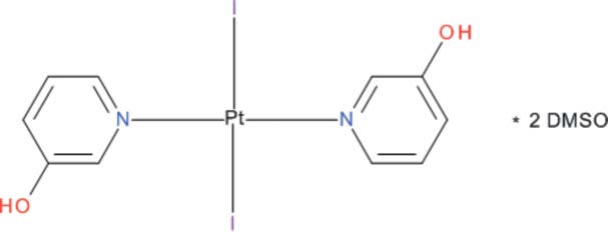


## Experimental

### 

#### Crystal data

[PtI_2_(C_5_H_5_NO)_2_]·2C_2_H_6_OS
*M* *_r_* = 795.35
Triclinic, 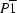

*a* = 6.0870 (12) Å
*b* = 7.8070 (16) Å
*c* = 12.305 (3) Å
α = 76.52 (3)°
β = 82.95 (3)°
γ = 81.87 (3)°
*V* = 560.5 (2) Å^3^
*Z* = 1
Mo *K*α radiation
μ = 9.22 mm^−1^
*T* = 293 K
0.19 × 0.15 × 0.05 mm

#### Data collection

Kuma KM-4 four-circle diffractometer
Absorption correction: analytical (*CrysAlis RED*; Oxford Diffraction, 2008) ▶ *T*_min_ = 0.091, *T*_max_ = 0.467
3570 measured reflections
3281 independent reflections
2568 reflections with *I* > 2σ(*I*)
*R*_int_ = 0.027
3 standard reflections every 200 reflections  intensity decay: 25.2%

#### Refinement

*R*[*F* ^2^ > 2σ(*F* ^2^)] = 0.037
*wR*(*F* ^2^) = 0.114
*S* = 1.07
3281 reflections
118 parameters
H-atom parameters constrained
Δρ_max_ = 1.59 e Å^−3^
Δρ_min_ = −2.75 e Å^−3^

### 

Data collection: *KM-4 Software* (Kuma, 1996 ▶); cell refinement: *KM-4 Software*; data reduction: *DATAPROC* (Kuma, 2001 ▶); program(s) used to solve structure: *SHELXS97* (Sheldrick, 2008 ▶); program(s) used to refine structure: *SHELXL97* (Sheldrick, 2008 ▶); molecular graphics: *SHELXTL* (Sheldrick, 2008 ▶); software used to prepare material for publication: *SHELXTL*.

## Supplementary Material

Crystal structure: contains datablocks I, global. DOI: 10.1107/S1600536811015893/lh5232sup1.cif

Structure factors: contains datablocks I. DOI: 10.1107/S1600536811015893/lh5232Isup2.hkl

Additional supplementary materials:  crystallographic information; 3D view; checkCIF report

## Figures and Tables

**Table 1:** Hydrogen-bond geometry (Å, °)

*D*—H⋯*A*	*D*—H	H⋯*A*	*D*⋯*A*	*D*—H⋯*A*
O1—H1⋯O2^i^	0.82	1.77	2.583 (7)	173

Symmetry code: (i) 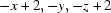.

**Fig. 1.**
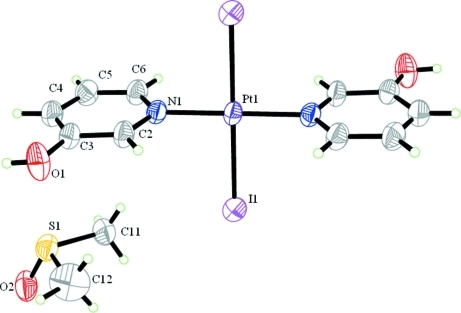
The labeled asymmetric unit and symmetry generated (-x+1, -y+1, -z+1) atoms of the complex molecule of the title compound with 50% probability displacement ellipsoids.

**Fig. 2.**
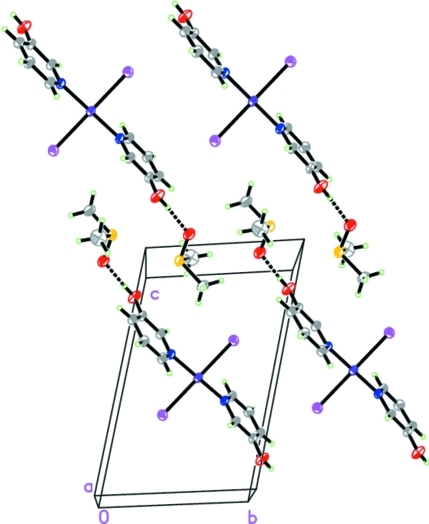
Part of the crystal structure with hydrogen bonds shown as dashed lines.

## supplementary crystallographic information

### Comment

Currently, attention is focused on platinum compounds that can bind to DNA differently than cisplatin with the idea that the different nature of binding with DNA may result into an altered spectrum of activity (Daghriri *et al.*, 2004). One such class of compounds are *trans*- planaramineplatinum complexes that bind with DNA to form mainly interstrand bifunctional 1,2-Pt(GG) adduct whereas cisplatin and its analogues form mainly intrastrand 1,2-Pt(GG) and 1,2-Pt(AG) adducts (Huq *et al.*, 2004). A number of *trans*-planaramineplatinum complexes have been prepared (Huq *et al.*, 2004; Chowdhury *et al.*, 2005; Beusichem & Farrell, 1992; Bierbach *et al.*, 1999; Farrell *et al.*, 1992). They have shown *in vitro* activity similar to cisplatin against various cancer cell lines. One of these compounds is *trans*-dichloro-bis(3-hydroxypyridine) platinum(II) (Huq *et al.*, 2004). In the title compound the chloride ligands have been replaced by iodide ligands. The crystal structure contains discrete molecules in which Pt^II^ ions lie on inversion centers (Fig. 1). Pt^II^ ions are coordinated to two symmetry related 3-hydroxypyridine ligand molecules *via* the pyridine N atoms and by two iodide ligands in a *trans* mode. The 3-hydroxypyridine ligand is planar with an r.m.s. of 0.0060 (2) Å. The coordination plane Pt/N1/I1/N1^i^/I1^i^ (Symmetry code: (i) -x+1, -y+1, -z+1) forms an angle of 72.8 (2)° with the ligand plane (N1/C2-C6/O1). In the crystal, complex molecules and solvent dimethyl sulfoxide molecules are linked by intermolecular O—H···O hydrogen bonds (Fig. 2).

### Experimental

1.0 mmol (415 mg) of K_2_PtCl_4_ was dissolved in 10 ml of ml water and 12 mmol (2.0 g) of KI was added and stirred for 30 min. 2.0 mmol (192 mg) of 3-hydroxypyridine, dissolved in 5 ml of ml water by sonification, was added with stirring to the mixture that was kept in ice. The mixture was stirred at room temperature for about 24 h. The yellow precipitate of Pt(3-hydroxypyridine)_2_I_2_ was collected by filtration, washed with ice cold water and ethanol, then air-dried. The precipitate was dissolved in a 1:1 DMSO:water mixture on heating and left standing. Crystals were obtained after 15 days.

### Refinement

The hydroxy group was included in the refinemnt with O-H = 0.82Å and *U*_iso_(H)= 1.2*U*_eq_(O). H atoms bonded to C atoms were placed in calculated positions with C—H = 0.93 and 0.96Å and treated as riding on the parent atoms with *U*_iso_(H)= 1.2*U*_eq_(C) or *U*_iso_(*H*)=1.5*U*_eq_(C_methyl_).

### Crystal data

**Table:**

[PtI_2_(C_5_H_5_NO)_2_]·2C_2_H_6_OS	*Z* = 1
*M**_r_* = 795.35	*F*(000) = 368
Triclinic, *P*1	*D*_x_ = 2.356 Mg m^−^^3^
Hall symbol: -P 1	Mo *K*α radiation, λ = 0.71073 Å
*a* = 6.0870 (12) Å	Cell parameters from 25 reflections
*b* = 7.8070 (16) Å	θ = 6–15°
*c* = 12.305 (3) Å	µ = 9.22 mm^−^^1^
α = 76.52 (3)°	*T* = 293 K
β = 82.95 (3)°	Plate, pale yellow
γ = 81.87 (3)°	0.19 × 0.15 × 0.05 mm
*V* = 560.5 (2) Å^3^	

### Data collection

**Table:**

Kuma KM-4 four-circle diffractometer	2568 reflections with *I* > 2σ(*I*)
Radiation source: fine-focus sealed tube	*R*_int_ = 0.027
graphite	θ_max_ = 30.1°, θ_min_ = 1.7°
profile data from ω/2θ scans	*h* = 0→8
Absorption correction: analytical (*CrysAlis RED*; Oxford Diffraction, 2008)	*k* = −10→10
*T*_min_ = 0.091, *T*_max_ = 0.467	*l* = −17→17
3570 measured reflections	3 standard reflections every 200 reflections
3281 independent reflections	intensity decay: 25.2%

### Refinement

**Table:**

Refinement on *F*^2^	Primary atom site location: structure-invariant direct methods
Least-squares matrix: full	Secondary atom site location: difference Fourier map
*R*[*F*^2^ > 2σ(*F*^2^)] = 0.037	Hydrogen site location: inferred from neighbouring sites
*wR*(*F*^2^) = 0.114	H-atom parameters constrained
*S* = 1.07	*w* = 1/[σ^2^(*F*_o_^2^) + (0.0739*P*)^2^ + 0.7284*P*] where *P* = (*F*_o_^2^ + 2*F*_c_^2^)/3
3281 reflections	(Δ/σ)_max_ < 0.001
118 parameters	Δρ_max_ = 1.59 e Å^−^^3^
0 restraints	Δρ_min_ = −2.75 e Å^−^^3^

### Special details

**Table:**

Geometry. All e.s.d.'s (except the e.s.d. in the dihedral angle between two l.s. planes) are estimated using the full covariance matrix. The cell e.s.d.'s are taken into account individually in the estimation of e.s.d.'s in distances, angles and torsion angles; correlations between e.s.d.'s in cell parameters are only used when they are defined by crystal symmetry. An approximate (isotropic) treatment of cell e.s.d.'s is used for estimating e.s.d.'s involving l.s. planes.
Refinement. Refinement of *F*^2^ against ALL reflections. The weighted *R*-factor *wR* and goodness of fit *S* are based on *F*^2^, conventional *R*-factors *R* are based on *F*, with *F* set to zero for negative *F*^2^. The threshold expression of *F*^2^ > σ(*F*^2^) is used only for calculating *R*-factors(gt) *etc*. and is not relevant to the choice of reflections for refinement. *R*-factors based on *F*^2^ are statistically about twice as large as those based on *F*, and *R*- factors based on ALL data will be even larger.

### Fractional atomic coordinates and isotropic or equivalent isotropic displacement parameters (Å^2^)

**Table:**

	*x*	*y*	*z*	*U*_iso_*/*U*_eq_	
Pt1	0.5000	0.5000	0.5000	0.03310 (11)	
I1	0.52440 (7)	0.68468 (5)	0.64805 (4)	0.04702 (13)	
S1	0.9980 (3)	0.2348 (2)	0.93997 (17)	0.0497 (4)	
N1	0.6905 (8)	0.2937 (6)	0.5856 (4)	0.0358 (9)	
O1	0.6176 (9)	−0.0245 (7)	0.8443 (5)	0.0581 (14)	
H1	0.7052	−0.0992	0.8805	0.087*	
O2	1.1336 (10)	0.2639 (7)	1.0279 (5)	0.0606 (14)	
C2	0.6050 (10)	0.1983 (8)	0.6833 (5)	0.0407 (12)	
H2	0.4573	0.2293	0.7080	0.049*	
C3	0.7252 (10)	0.0565 (7)	0.7490 (5)	0.0385 (11)	
C6	0.9025 (10)	0.2500 (8)	0.5501 (6)	0.0425 (13)	
H6	0.9629	0.3151	0.4826	0.051*	
C4	0.9432 (11)	0.0105 (8)	0.7117 (6)	0.0449 (13)	
H4	1.0283	−0.0857	0.7528	0.054*	
C5	1.0354 (11)	0.1105 (9)	0.6109 (6)	0.0470 (14)	
H5	1.1835	0.0837	0.5852	0.056*	
C11	1.0277 (16)	0.4169 (11)	0.8257 (7)	0.063 (2)	
H11A	0.9749	0.5250	0.8500	0.095*	
H11B	0.9423	0.4073	0.7673	0.095*	
H11C	1.1819	0.4176	0.7978	0.095*	
C12	0.7179 (15)	0.2967 (17)	0.9862 (10)	0.087 (3)	
H12A	0.6764	0.2172	1.0555	0.130*	
H12B	0.6230	0.2911	0.9306	0.130*	
H12C	0.7020	0.4156	0.9976	0.130*	

### Atomic displacement parameters (Å^2^)

**Table:**

	*U*^11^	*U*^22^	*U*^33^	*U*^12^	*U*^13^	*U*^23^
Pt1	0.02968 (15)	0.02872 (14)	0.03610 (16)	0.00271 (9)	−0.00194 (10)	−0.00172 (10)
I1	0.0506 (2)	0.0420 (2)	0.0489 (3)	0.00216 (18)	−0.00824 (19)	−0.01362 (18)
S1	0.0558 (9)	0.0362 (7)	0.0553 (10)	−0.0041 (6)	−0.0098 (8)	−0.0051 (7)
N1	0.039 (2)	0.0284 (19)	0.036 (2)	0.0004 (17)	−0.0004 (18)	−0.0029 (17)
O1	0.048 (3)	0.055 (3)	0.057 (3)	−0.006 (2)	−0.005 (2)	0.017 (2)
O2	0.070 (3)	0.047 (3)	0.063 (3)	−0.002 (2)	−0.027 (3)	0.000 (2)
C2	0.035 (3)	0.037 (3)	0.045 (3)	0.006 (2)	−0.006 (2)	−0.003 (2)
C3	0.040 (3)	0.030 (2)	0.042 (3)	−0.003 (2)	−0.003 (2)	−0.002 (2)
C6	0.037 (3)	0.040 (3)	0.046 (3)	0.004 (2)	−0.001 (2)	−0.006 (2)
C4	0.044 (3)	0.040 (3)	0.048 (3)	0.004 (2)	−0.012 (3)	−0.007 (3)
C5	0.036 (3)	0.050 (3)	0.051 (4)	0.008 (2)	−0.004 (2)	−0.011 (3)
C11	0.082 (6)	0.053 (4)	0.045 (4)	0.006 (4)	−0.003 (4)	0.000 (3)
C12	0.052 (5)	0.111 (8)	0.099 (8)	−0.030 (5)	0.011 (5)	−0.024 (7)

### Geometric parameters (Å, °)

**Table:**

Pt1—N1^i^	2.007 (5)	C3—C4	1.376 (9)
Pt1—N1	2.007 (5)	C6—C5	1.385 (8)
Pt1—I1	2.6021 (8)	C6—H6	0.9300
Pt1—I1^i^	2.6021 (8)	C4—C5	1.402 (10)
S1—O2	1.514 (6)	C4—H4	0.9300
S1—C11	1.763 (8)	C5—H5	0.9300
S1—C12	1.767 (10)	C11—H11A	0.9600
N1—C6	1.334 (7)	C11—H11B	0.9600
N1—C2	1.345 (8)	C11—H11C	0.9600
O1—C3	1.336 (8)	C12—H12A	0.9600
O1—H1	0.8200	C12—H12B	0.9600
C2—C3	1.383 (8)	C12—H12C	0.9600
C2—H2	0.9300		
			
N1^i^—Pt1—N1	179.999 (1)	N1—C6—H6	119.0
N1^i^—Pt1—I1	89.13 (15)	C5—C6—H6	119.0
N1—Pt1—I1	90.87 (15)	C3—C4—C5	119.1 (6)
N1^i^—Pt1—I1^i^	90.87 (15)	C3—C4—H4	120.5
N1—Pt1—I1^i^	89.13 (15)	C5—C4—H4	120.5
I1—Pt1—I1^i^	180.0	C6—C5—C4	119.0 (6)
O2—S1—C11	105.5 (4)	C6—C5—H5	120.5
O2—S1—C12	105.1 (5)	C4—C5—H5	120.5
C11—S1—C12	97.6 (5)	S1—C11—H11A	109.5
C6—N1—C2	118.5 (5)	S1—C11—H11B	109.5
C6—N1—Pt1	122.1 (4)	H11A—C11—H11B	109.5
C2—N1—Pt1	119.5 (4)	S1—C11—H11C	109.5
C3—O1—H1	109.5	H11A—C11—H11C	109.5
N1—C2—C3	123.5 (5)	H11B—C11—H11C	109.5
N1—C2—H2	118.3	S1—C12—H12A	109.5
C3—C2—H2	118.3	S1—C12—H12B	109.5
O1—C3—C4	125.5 (5)	H12A—C12—H12B	109.5
O1—C3—C2	116.5 (6)	S1—C12—H12C	109.5
C4—C3—C2	118.0 (6)	H12A—C12—H12C	109.5
N1—C6—C5	121.9 (6)	H12B—C12—H12C	109.5

Symmetry codes: (i) −*x*+1, −*y*+1, −*z*+1.

### Hydrogen-bond geometry (Å, °)

**Table:**

*D*—H···*A*	*D*—H	H···*A*	*D*···*A*	*D*—H···*A*
O1—H1···O2^ii^	0.82	1.77	2.583 (7)	173

Symmetry codes: (ii) −*x*+2, −*y*, −*z*+2.
